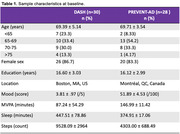# The Temporal Relationship Between Physical Activity, Mood, and Sleep in Older Adults: Lead‐Lag Discovery and Replication Analyses

**DOI:** 10.1002/alz.093480

**Published:** 2025-01-09

**Authors:** Ryan Kara, Adrian Noriega de la Colina, Meishan Ai, Shania Fock Ka Bao, Arthur Kramer, Jennifer Tremblay‐Mercier, Sylvia Villeneuve, Linda Li, Caitlin Walker, Maiya R. Geddes

**Affiliations:** ^1^ The Montreal Neurological Institute, McGill University, Montréal, QC Canada; ^2^ The Montreal Neurological Institute, McGill University, Montreal, QC Canada; ^3^ Douglas Mental Health University Institute, Montreal, QC Canada; ^4^ Northeastern University, Boston, MA USA; ^5^ McGill University, Montreal, QC Canada; ^6^ Douglas Hospital Research Centre, Montreal, QC Canada; ^7^ Centre for Studies on Prevention of Alzheimer’s Disease (StoP‐AD Centre), Montreal, QC Canada; ^8^ Centre for Studies on Prevention of Alzheimer’s disease (StoP‐AD Centre), Douglas Mental Health Institute, Montreal, QC Canada; ^9^ Douglas Mental Health Research Centre, Montreal, QC Canada; ^10^ StoP‐AD Centre, Douglas Mental Health Institute Research Centre, Montreal, QC Canada; ^11^ Montreal Neurological Institute, McGill University, Montréal, QC Canada

## Abstract

**Background:**

Alzheimer’s disease (AD) affects about 416 million individuals across the disease continuum. An estimated 40% of dementia cases can be prevented or delayed in onset by addressing modifiable risk factors like sleep time, physical activity (PA), and mood. These three behaviors (sleep time, physical inactivity, and mood) are linked to cognitive decline, and their tridirectional link has been shown by prior research work. However, their longitudinal and potential temporal interrelatedness remain unclear.

**Method:**

Thirty cognitively unimpaired (CU) sedentary older adults participating in the Daily Activity Study of Health (DASH) (Clinicaltrials.gov:NCT04315363) underwent objective PA measurement using a wrist‐worn accelerometer to monitor 24‐hour daily activity (discovery sample). Participants also completed a daily morning mood scale; through an Ecological Momentary Assessment and daily activity data, we performed a 10‐day lead‐lag analysis using moderate‐to‐vigorous PA (MVPA) time, mood, and sleep time. The temporal patterns were derived by assessing the rank order of the lead‐lag coefficients. The relationship between different time points of the variables were examined through cross‐correlations. We replicated the results in a sample of 25 CU, physically inactive older adults from the Healthy Aging Brain Study (HABS) (Clinical trials.gov:NCT06038643) with familial risk of AD. An identical methodological and analytic approach was applied to this replication sample.

**Result:**

The lead‐lag analysis in the DASH cohort (mean age in years: 69.39±5.14, 26 female, mean education years 16.60 ± 3.03) determined positive mood change preceded increase in sleep time, and was then followed by an increase in MVPA. The relationship between positive mood change and increased MVPA was replicated in the HABS lead‐lag analysis (mean age = 69.71±3.54 years; 20 females; mean education years = 16.12±2.99).

**Conclusion:**

In this study, we found that (1) improved mood precedes the increase in physical activity and sleep time in CU sedentary older adults. We confirmed that MVPA and mood precede sleep time. We also found that (2) mood precedes MVPA time, which in turn precedes sleep time. Understanding temporal dynamics between mood, sleep time, and MVPA, can help develop better interventions targeting an increase in physical inactivity in older adults, a major risk factor of AD.